# Clinical and antibody characteristics reveal diverse signatures of severe and non-severe SARS-CoV-2 patients

**DOI:** 10.1186/s40249-022-00940-w

**Published:** 2022-02-02

**Authors:** Hongye Wang, Dongshan Yan, Ya Li, Yanfei Gong, Yulin Mai, Bingxiang Li, Xiaoyong Zhu, Xinrui Wan, Liyun Xie, HuaKe Jiang, Min Zhang, Ming Sun, Yufeng Yao, Yongzhang Zhu

**Affiliations:** 1 Institute of Medical Biology, Chinese Academy of Medical Sciences and Peking Union Medical College, Kunming, 650118 Yunnan China; 2 School of Basic Medical Science, Kunming Medical University, Kunming, 650500 China; 3 Yunnan Key Laboratory of Laboratory Medicine, Yunnan Province Clinical Research Center for Laboratory Medicine, Yunnan Innovation Team of Clinical Laboratory and Diagnosis, First Affiliated Hospital of Kunming Medical University, Kunming, 650032 China; 4 Medical Examination Center, The First People’s Hospital of Yueyang City, Yueyang, 414000 Hunan China; 5 Department of Medicine, Peking Union Medical College Hospital, Peking Union Medical College and Chinese Academy of Medical Sciences, Beijing, 100730 China; 6 School of Global Health, Chinese Center for Tropical Diseases Research, Shanghai Jiao Tong University School of Medicine; One Health Center, Shanghai Jiao Tong University-The University of Edinburgh, Shanghai, 200025 China

**Keywords:** SARS-CoV-2, COVID-19, Severe patient, Cytokine, Immune response

## Abstract

**Background:**

COVID-19 pandemic continues, clarifying signatures in clinical characters and antibody responses between severe and non-severe COVID-19 cases would benefit the prognosis and treatment.

**Methods:**

In this study, 119 serum samples from 37 severe or non-severe COVID-19 patients from the First People's Hospital of Yueyang were collected between January 25 and February 18 2020. The clinical features, antibody responses targeting SARS-CoV-2 spike protein (S) and its different domains, SARS-CoV-2-specific Ig isotypes, IgG subclasses, ACE2 competitive antibodies, binding titers with FcγIIa and FcγIIb receptors, and 14 cytokines were comprehensively investigated. The differences between severe and non-severe groups were analyzed using Mann–Whitney U test or Fisher’s exact test.

**Results:**

Severe group including 9 patients represented lower lymphocyte count, higher neutrophil count, higher level of LDH, total bile acid (TBA) (*P* < 1 × 10^–4^), r-glutaminase (*P* = 0.011), adenosine deaminase (*P* < 1 × 10^–4^), procalcitonin (*P* = 0.004), C-reactive protein (*P* < 1 × 10^–4^) and D-dimer (*P* = 0.049) compared to non-severe group (28 patients). Significantly, higher-level Igs targeting S, different S domains (RBD, RBM, NTD, and CTD), FcγRIIa and FcγRIIb binding capability were observed in a severe group than that of a non-severe group, of which IgG1 and IgG3 were the main IgG subclasses. RBD-IgG were strongly correlated with S-IgG both in severe and non-severe group. Additionally, CTD-IgG was strongly correlated with S-IgG in a non-severe group. Positive RBD-ACE2 binding inhibition was strongly associated with high titers of antibody (S-IgG1, S-IgG3, NTD-IgG, RBD-IgA, NTD-IgA, and CTD-IgA) especially RBD-IgG and CTD-IgG in the severe group, while in the non-severe group, S-IgG3, RBD-IgG, NTD-IgG, and NTD-IgM were correlated with ACE2 blocking rate. S-IgG1, NTD-IgM and S-IgM were negatively associated with illness day in a severe group, while S-IgG3, RBD-IgA, CTD-IgA in the severe group (*r* = 0.363, *P* = 0.011) and S-IgG1, NTD-IgA, CTD-IgA in the non-severe group were positively associated with illness day. Moreover, GRO-α, IL-6, IL-8, IP-10, MCP-1, MCP-3, MIG, and BAFF were also significantly elevated in the severe group.

**Conclusion:**

Antibody detection provides important clinical information in the COVID-19 process. The different signatures in Ig isotypes, IgG subclasses, antibody specificity between the COVID-19 severe and non-severe group will contribute to future therapeutic and preventive measures development.

**Graphical Abstract:**

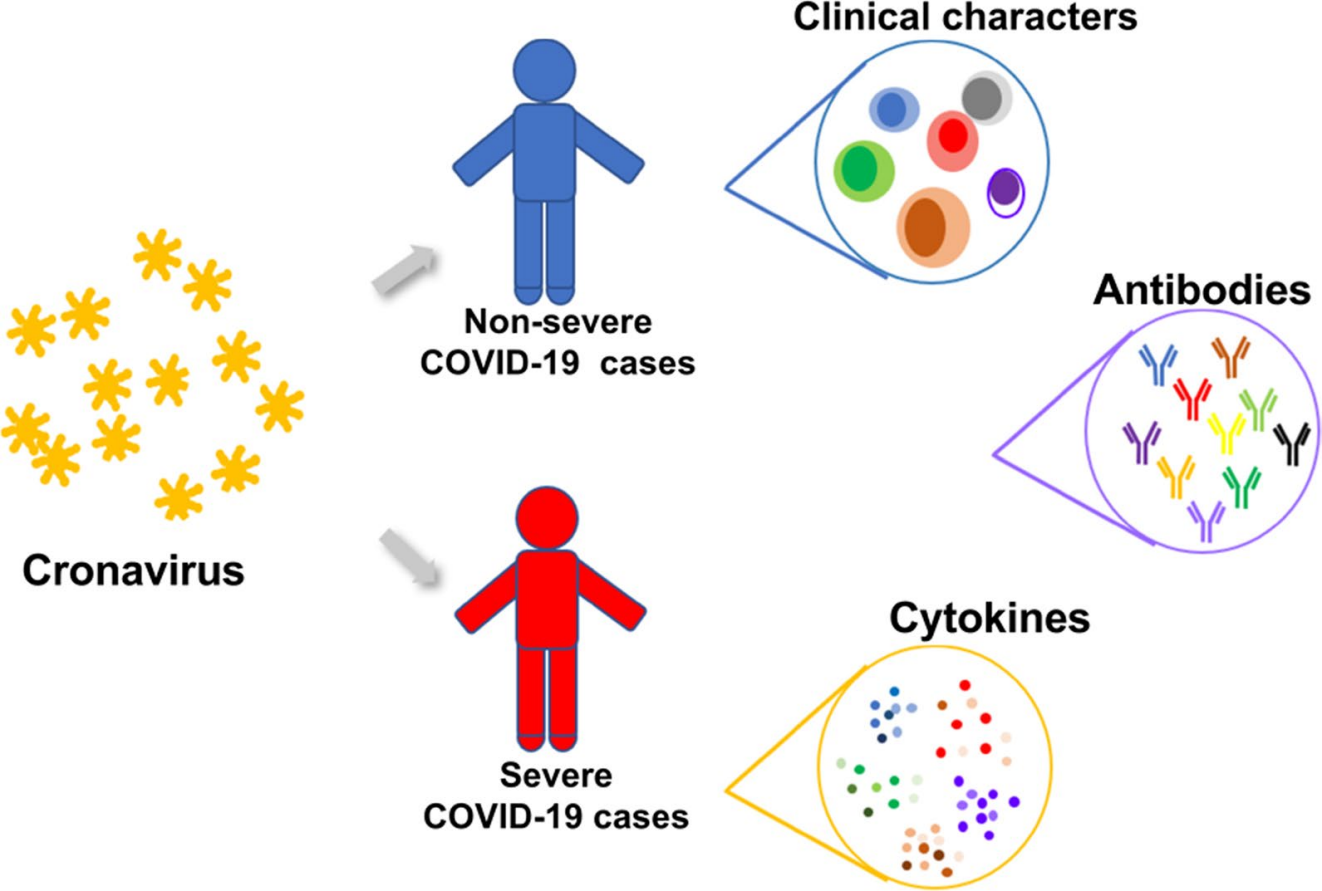

**Supplementary Information:**

The online version contains supplementary material available at 10.1186/s40249-022-00940-w.

## Background

The coronavirus disease 2019 (COVID-19), caused by severe acute respiratory syndrome coronavirus 2 (SARS-CoV-2) [1, 2], has been declared a threat to global health. It has caused over 300 million COVID-2019 cases and accounting for over 5 million deaths [3].

Similar to SARS-CoV infection, the common clinical manifestations of COVID-19 include fever, cough, fatigue, sore throat, dyspnea and pneumonia, with low total lymphocyte count and percentage of T cells, increased C-reactive protein (CRP) concentration and erythrocyte sedimentation rate [4]. According to the clinical severity, the COVID-19 cases can be divided into mild, moderate, or severe subtypes. Severe cases are defined by respiratory distress with pneumonia, with respiratory rate ≥ 30 breaths/min; or SpO_2_ (oxygen saturation) ≤ 93% at rest; or PaO_2_/FIO_2_ (partial pressure of oxygen/fraction of inspired oxygen) ≤ 300 mmHg. It is reported that dyspnea, myalgia or fatigue, high-grade fever were the most common symptoms in severe cases. CRP, lactate dehydrogenase (LDH) and D-dimer level in severe cases were significantly higher than mild or moderate patients [5, 6]. Differences in clinical manifestations were primarily due to individual immune response, especially antibody titers.

Antibody plays an important role of humoral response after microbial infection. There are five antibody isotypes in serum, including IgA, IgD, IgE, IgM, and IgG. Following SARS-CoV-2 infection, virus-specific IgM, IgG, and IgA antibody have been detected [7, 8], of which IgG is the most abundant. The infection of SARS-CoV-2 relies on the interaction between the receptor binding domain (RBD) of its spike protein (S) and the angiotensin converting enzyme 2 (ACE2) on host cells [9, 10]. Multiple studies have shown that the majority of SARS-CoV-2-infected individuals produce S- and RBD-specific antibodies [11, 12]. In addition, other studies also reported isolation of N-terminal domain (NTD)-specific and S2-specific monoclonal antibodies that exhibited high neutralization potency [13, 14]. However, detailed information on antibody targeting domain of the spike protein and the frequency of the antibody was not clarified clearly.

Despite the importance of antibody protection, concerns of antibody-dependent enhancement (ADE) arise from the possibility that existing antibody may increase the severity of disease, which may be caused by antibody-mediated endocytosis into Fc gamma receptor IIa (FcγRIIa)-expressing phagocytic cells, leading to rapid viral replication. Several studies have reported increased uptake of SARS-CoV and Middle East Respiratory Syndrome Coronavirus virions into FcR-expressing monocytes or macrophages in vitro [15, 16]. However, FcγRIIb, the only inhibitory Fc receptor that cross-links with the activated receptor to intracellular transduction inhibitory signals, played a significant role in the negative regulation of immune response.

Besides the antiviral effect of antibodies, cytokines are also important components in an antiviral immune response. The proliferation of immune cells and signal factors lead to local inflammation and even cytokine storm syndrome (CSS). In COVID-19 patients, the studies reported elevated interleukins (IL) like IL-6, IL-8, IL-2R, IL-10, tumor necrosis factor (TNF-α), IL-1Ra, IP-10 (IFN-γ-induced protein 10) and macrophage inflammatory protein 1 (MCP-1) [17–21], especially in the severe group.

Further comparison of differences in cytokines and immune characters between severe and non-severe patients will help better to clarify the relationship between inflammation and antibody responses. Thus, in the present study, we characterized the clinical and immune features of 119 blood samples collected from 37 hospitalized patients with mild to severe symptoms, focusing on antibody isotype and IgG titers, RBD-ACE2 blocking activity, binding tiers with FcγR, B cell activation factor and cytokines. We carefully compared how these responses differentiated between the severe group and the non-severe group. Finally, the interplay between antibody isotype, antibody subclasses, antibody dynamics and functional antibody characteristics were analyzed in detail to provide the full understanding of host immune response against SARS-CoV-2 infection between the severe group and non-severe group.

## Methods and materials

### Study samples

Serum samples were collected from 37 COVID-19 patients at the First People's Hospital of Yueyang between January 25 and February 18 2020. All individuals had PCR-confirmed SARS-CoV-2 infection and related symptoms. These patients were divided into a severe and non-severe (mild or moderate) group. Nine were classified severe (severe group), while 28 were mild or moderate (non-severe group). The cohort included 21 females and 16 males, with a median age of 53.5 (25–75) years. Thirty-seven COVID-19 patients were serially sampled during the hospitalization, and a total of 119 serum samples were finally collected. The serum samples were heat inactivated at 56 ℃ for 30 min before use.

### Proteins

The proteins used in this study were purchased or customized from Sino Biological (Beijing, China) or Novoprotein (Shanghai, China), and detailed information was listed in Additional file 2: Table S2.

### Measurements of SARS-CoV-2-specific antibodies

Antibody responses, mainly target the spike proteins, which make it important to evaluate S-specific antibody responses. Using an in-house enzyme-linked immunosorbent assay (ELISA), we measured the presence of anti-SARS-CoV-2 antibody isotypes and IgG subtypes. ELISA was used to measure the SARS-CoV-2-specific IgG, IgM, IgA, and subclasses of IgG (IgG1–G4). 1 μg/ml of the recombinant S, RBD, RBM, NTD, or CTD proteins in phosphate-buffered saline (PBS) (pH 7.4) were used to coat the 96-well plates at 4 °C overnight. Plates were washed with phosphate-buffered saline, 0.05% Tween-20 (PBST) five times after each binding step. Plates were blocked with blocking buffer (PBS containing 5% BSA) at 37 °C for 2 h. The serum samples diluted in PBS containing 1% BSA at 1:100 was added to plates for screening assay, and serially diluted serum samples staring from 100-fold dilution were added to plates for binding titer test. The bound antibodies were detected with horseradish peroxidase (HRP)-conjugated goat anti-human IgG, IgM, and IgA (1:10,000, Abcam) and mouse anti-human IgG1, IgG2 (1:1,000, Abcam), IgG3 (1:1,000, Thermo Fisher, USA), and IgG4 (1:4,000, Abcam). The plates were then washed five times and incubated with TMB substrate (Solarbio, Beijing, China) at room temperature for 15 min, and a stop solution (Solarbio, Beijing, China) was then added. The absorbance at 450 nm (OD_450_) was measured using an ELISA microplate reader (Molecular Devices, Sunnyvale, USA). Absorbance values at 650 nm (OD_650_) were also measured and subtracted to eliminate the background color and absorbance value of the pore plate itself. Each sample was tested in duplicate, and the results are reported as the mean values.

### ACE2 blocking assay

To test the effect of serum on blocking ACE2 binding RBD, 2 μg/ml the recombinant ACE2 (Sino iological, Beijing, China) was added in 96-well plates and overnight at 4 °C, followed by blocking with the blocking buffer and washing. RBD-mouse-Ig-Fc at a concentration of 0.15 μg/ml was pre-incubated with serum diluted at 1:20 at 37 °C for 1 h, and then added into the wells coated with ACE2 and incubated at 37 °C for 1 h. Then the proportion of RBD-Fc proteins that were blocked by serum could not bind with ACE2 and were washed away. Goat anti-mouse IgG antibodies were added and incubated at 37 °C for 1 h, followed by adding TMB substrates and incubated at room temperature for 15 min. Stop solution was added and measured as above. The blocking percentage were calculated 100 × (1−(OD_450_ value of serum sample/OD_450_ value of PBS control)). Each sample was tested in duplicate, and the results are reported as the mean values.

### Cytokine measurements

ELISA was used to measure the serum levels of APRIL (BioLegend, San Diego, USA) and BAFF (R&D Systems, Minneapolis, USA) according to the manufacturer’s instructions. Serum cytokines (GRO-α, IFN-γ, IL-1β, IL-1-ra, IL-6, IL-8, IL-15, IP-10, MCP-1, MCP-3, MIG, and VEGFA) were measured with a multiplex assay (Human Cytokine/Chemokine Panel I, Millipore, Billerica, USA) on a Luminex200 platform. Each sample was tested in duplicate, and the results are reported as the mean values.

### Statistical analyses

All the continuous variables and categorical variables in this study were expressed as median [interquartile range (IQR)] and number/sum (%). Differences in continuous variables between severe group and non-severe groups were compared using Mann–Whitney U test. Fisher’s exact test was used to analyze two-group categorical variables. The correlations were determined by the Spearman rank method. *P* values < 0.05 and *r* > 0.3 or < − 0.3 were considered statistically significant. *P* values between 0.01 and 0.05, 0.001 and 0.01, 0.0001 and 0.001, and < 0.0001 were considered statistically significant (*), very significant (**), extremely significant (***) and super significant (****), respectively, whereas “ns” represents not significant. The analyses were performed using GraphPad Prism 9 software (GraphPad, La Jolla, California, USA).

## Results

### Demographic and clinical features

A total of 37 COVID-19 patients were included in the current study, including 21 females and 16 males. These patients were divided into a severe and non-severe (mild or moderate) group based on the disease severity. The median age of the patients was 53.5 years, ranging from 27 to 76 years. There were no significant differences in age and gender between the two groups (Additional file 1: Table S1). A total of 119 serum samples from the 37 patients were serially collected, ranging from 6 days after symptom onset to 45 days during hospitalization. The median sample days in severe and non-severe group were 18.5 and 19 days after symptoms onset.

For clinical manifestations, common symptoms in our cohort included fever, cough, fatigue, sore throat and chest tightness (Additional file 1: Table S1). High grade fever (*P* < 1 × 10^–4^), chest tightness (*P* = 0.007), shortness of breath (*P* = 6 × 10^–4^), nausea or vomiting (*P* = 9 × 10^–4^) were reported significantly more in severe group compared to non-severe group. Severe group also had more comorbidities such as diabetes (*P* = 1.9 × 10^–4^) (Additional file 1: Table S1).

As shown in Table 1, blood examination results showed that both absolute count and percentage of leukocyte and neutrophil were significantly higher in a severe group than non-severe group (*P* < 0.05), while the percentage of lymphocyte and monocyte were significantly lower in the severe group. Serum biochemical study showed that the severe cases had significantly higher levels of LDH (*P* = 0.005), total bile acid (TBA) (*P* < 1 × 10^–4^), r-glutaminase (*P* = 0.011), adenosine deaminase (*P* < 1 × 10^–4^), procalcitonin (*P* = 0.034), CRP (*P* < 1 × 10^–4^) and D-dimer (*P* = 0.050) compared to non-severe cases (Table 1). Lower percentage of CD3^+^ T cell, CD3^−^CD16/56^+^ NK cell and higher CD3^−^CD19^+^ B cell percentage in severe groups were also observed, but due to the limited flow cytometry analysis data (severe case *n* = 7, non-severe case *n* = 17), the differences were not statistically significant (*P* > 0.05). These results suggested increased systemic inflammation, dysfunction of the liver, and compromised T cell response is associated with the severity of COVID-19 patients.

**Table 1:** Laboratory findings in COVID-19 patients

Laboratory items	Normal range	All patients (*n* = 37)	Severe (*n* = 9)	Non-severe (*n* = 28)	*P* value
White blood cell (× 10^9^/L)	3.5–9.5	5.61 (4.21–10.21)	11.40 (7.20–15.59)	5.45 (4.13–7.11)	0.001
Neutrophil (× 10^9^/L)	1.8–6.3	3.58 (2.39–8.07)	10.81(5.17–13.29)	3.30 (2.35–5.20)	5 × 10^–4^
Lymphocyte (× 10^9^/L)	1.1–3.2	1.02 (0.68–1.70)	0.78 (0.46–0.96)	1.22 (0.89–1.83)	0.053
Monocyte (× 10^9^/L)	0.1–0.6	0.54 (0.34–0.73)	0.78 (0.10–1.33)	0.54 (0.35–0.70)	0.043
Neutrophil (%)	40–75	65.40 (56.40–84.20)	84.20 (74.20–87.95)	60.80 (54.98–76.43)	0.002
Lymphocyte (%)	20–50	23.00 (11.05–31.20)	11.20 (5.75–18.95)	25.10 (14.23–32.03)	0.005
Monocyte (%)	3–10	8.50 (6.05–11.00)	6.00 (2.40–8.45)	9.60 (7.33–11.78)	0.005
Lactate dehydrogenase (U/L)	120–250	178.0 (155.0–208.7)	334.7 (191.0–476.5)	163.6 (149.4–185.5)	0.005
Alkaline Phosphatase (U/L)	45–125	61.70 (46.85–72.45)	59.13 (42.90–61.95)	68.15 (48.50–73.28)	0.091
Total bile acid (μmol/L)	0–12	4.15 (2.15–5.92)	5.48 (2.33–6.99)	0.80 (2.07–5.65)	< 1 × 10^–4^
r-glutaminase (U/L)	10–60	28.28 (14.90–85.74)	100.10 (23.25–187.60)	27.25 (11.98–43.60)	0.011
Adenosine deaminase (U/L)	4–24	9.22 (8.04–11.47)	12.04 (10.11–15.70)	8.87 (7.87–10.11)	< 1 × 10^–4^
Procalcitonin (ng/ml)	< 0.046	0.10 (0.04–0.70)	0.80 (0.42–3.12)	0.06 (0.04–0.10)	0.034
C-reactive protein (mg/L)	0–10	25.37 (1.97–58.70)	70.13 (58.70–168.50)	8.39 (1.70–33.47)	< 1 × 10^–4^
D-dimer (ng/ml)	< 0.5	340.3 (175.0–485.0)	416.0 (350.0–910.0)	290.0 (150.0–445.0)	0.050
CD3^+^T (%)	50–84	73.67 (60.61–79.61)	65.23 (56.16–75.80)	75.05 (62.44–80.84)	0.312
CD3^+^T cell count	955–2,860	887.0 (586.3–1,509.0)	991.0 (618.0–1,216.0)	880.0 (571.5–1,553.0)	0.952
CD3^+^CD4^+^T (%)	27–51	41.31 (31.04–49.54)	47.71 (28.48–50.06)	41.03 (32.04–49.06)	0.716
CD^3+^CD4^+^T cell count	550–1,440	501.0 (335.8–673.8)	612.0 (313.0–682.0)	499.0 (353.5–647.5)	0.479
CD3^+^CD8^+^T (%)	15–44	24.41 (17.10–30.37)	24.41 (17.10–30.37)	21.56 (17.59–31.49)	0.899
CD3^+^CD8^+^T cell count	320–1,250	319.5 (192.5–523.0)	338.0 (301.0–540.0)	282.0 (170.0–512.0)	0.884
CD3^−^CD16/56^+^ NK (%)	7–40	12.48 (8.78–17.87)	9.37 (5.55–30.43)	13.06 (9.67–17.29)	0.809
CD3^−^CD16/56^+^ cell count	150–1,100	174.5 (121.3–226.0)	164.0 (76.0–335.0)	185.0 (133.5–219.5)	0.554
CD3^−^CD19^+^ B (%)	5–18	11.63 (8.02–17.50)	18.36 (11.57–22.38)	11.40 (7.51–13.70)	0.089
CD3^−^CD19^+^ B cell count	90–560	141.0 (97.5–300.8)	250.0 (127.0–354.0)	107.0 (96.0–259.5)	0.123

### Anti-SARS-CoV-2 antibody responses

**Fig. 1:**
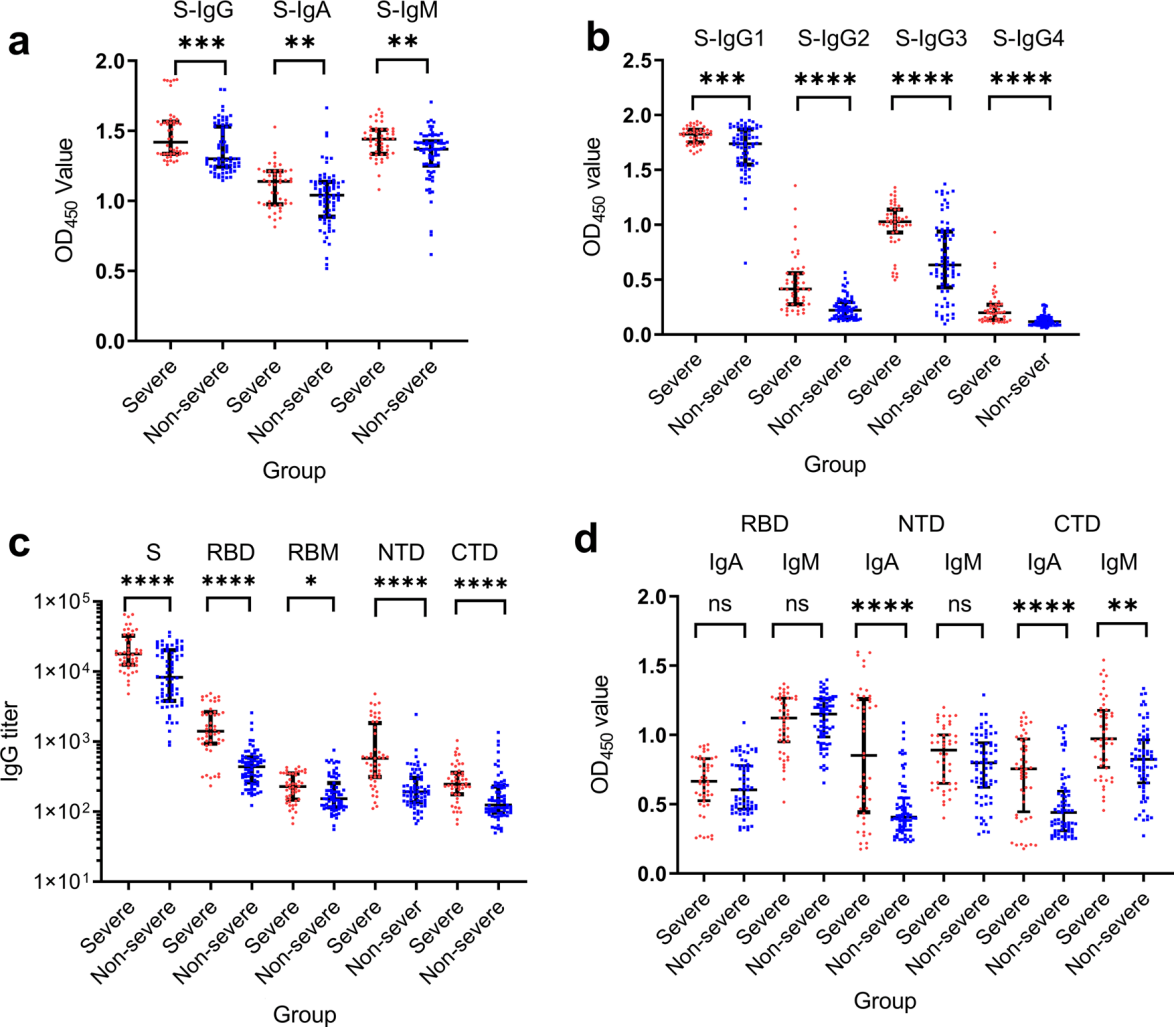
SARS-CoV-2 specific antibodies in COVID-19 severe cases and non-severe cases. Serum samples from severe cases (*n* = 48) and non-severe cases (*n* = 71) were compared for SARS-CoV-2 S specific antibody isotypes: IgG, IgA, and IgM (**a**), different anti-S IgG subtypes (IgG1, IgG2, IgG3, and IgG4) (**b**), IgG titers targeting S, RBD, RBM, NTD, and CTD (**c**), IgA and IgM response targeting RBD, NTD, and CTD (**d**). The OD_450_ values were normalized by subtracting OD_650_ values. The antibody titers were the dilution fold that reached half-maximal binding with corresponding antigens, and the values were calculated by Graphpad Prism 9. Mann–Whitney U test was used to compare differences between the two groups. Significances were marked as follows: *P* < 0.05 (*), *P* < 0.01(**), *P* < 0.001 (***), and *P* < 0.0001 (****), respectively. Abbreviations: S: Spike; RBD: Receptor Binding Domain; NTD: N-terminal Domain; CTD: C-terminal Domain

Serum anti-SARS-CoV-2 S-specific IgG, IgA, and IgM antibodies were detected in all samples (Fig. 1a), and antibody levels in the severe group were all significantly higher than the non-severe group (*P* < 0.001). The four IgG subclasses targeting SARS-CoV-2 S protein were detected in all samples, with overall IgG1 and IgG3 responses higher than IgG2 and IgG4 responses. The severe group also showed higher IgG1-IgG4 levels than the non-severe group (*P* < 1 × 10^–4^) (Fig. 1b). Serum IgG titers against SARS-CoV-2 S, RBD, receptor binding motif (RBM), N terminal domain (NTD), and C-terminal domain (CTD) were measured by ELISA. S-targeting IgG titer in the severe group ranged from 4,818 to 65,392 (median 17,803), followed by RBD-specific IgG titers (233–ؘ4,871, median 1,406), NTD-specific IgG titers (111–4,795, median 579), CTD-specific IgG titers (66–1,038, median 247), and RBM-specific IgG titers were the lowest (67–438, median 228) (Fig. 1c). A similar trend of IgG titers was observed in the non-severe group. As expected, the S-targeting IgG titer is the highest, ranging from 889 to 36,571 (median 8,282), followed by RBD-specific IgG titers (123–2,574, median 437), NTD-specific IgG titers (67–2,448, median 192), CTD-specific IgG titers (50–1,353, median 125), and RBM-specific IgG titers were the lowest (55–754, median 153). The S-targeting IgG titers and different S-domain-targeting IgG titers were also significantly higher in the severe group (*P* < 0.0001 except for RBM, of which *P* = 0.013) than the non-severe group. Furthermore, IgA and IgM responsible to target different domains of S (RBD, NTD, and CTD) were also compared (Fig. 1d). Significantly higher NTD-targeting IgA (*P* < 1 × 10^–4^), CTD-targeting IgA (*P* < 1 × 10^–4^), and CTD-targeting IgM (*P* = 0.001) were found in the severe group than the non-severe group. The results indicated an overall higher antibody response in the severe COVID-19 infection group.

### Serum antibody blocking RBD binding to ACE2

**Fig. 2:**
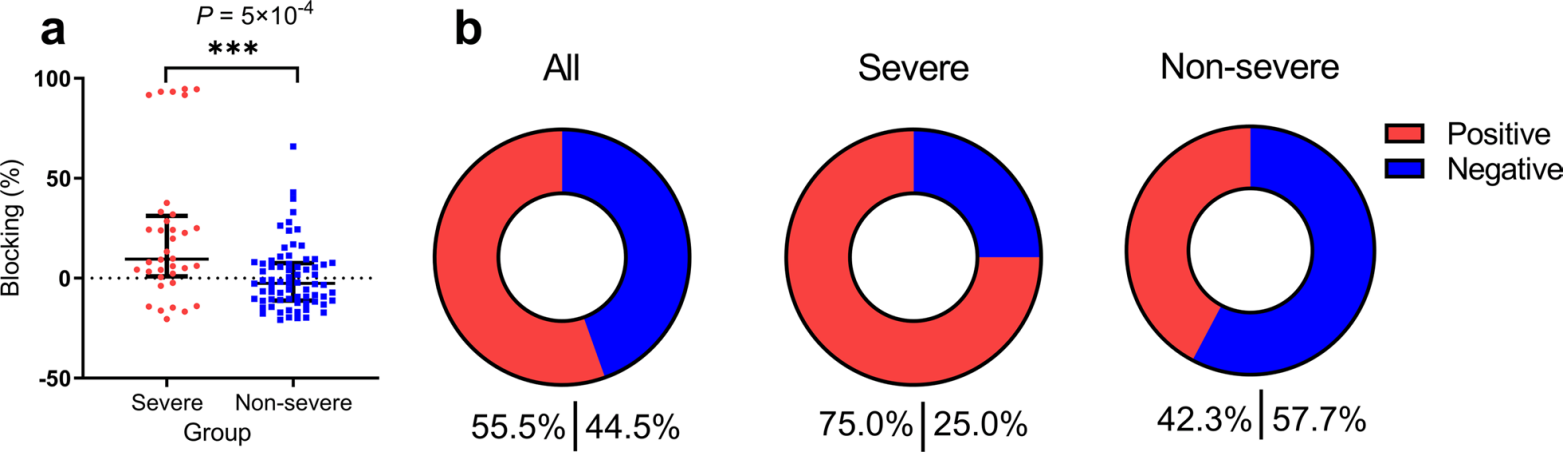
Comparison of RBD–ACE2 binding inhibition of serum samples between the severe and non-severe group. **a** The blocking percentage of serum to inhibit RBD-ACE2 interaction were showed. Serum was diluted at a final dilution of 1:40. The blocking percentages were calculated as 100 × (1 − (OD_450_ value of serum sample/OD_450_ value of PBS control)). **b** Pie charts showing the proportions of samples with positive (Red) or negative (Blue) RBD-ACE2-binding inhibition. Mann–Whitney U test was used to compare differences between the two groups

**Fig. 3:**
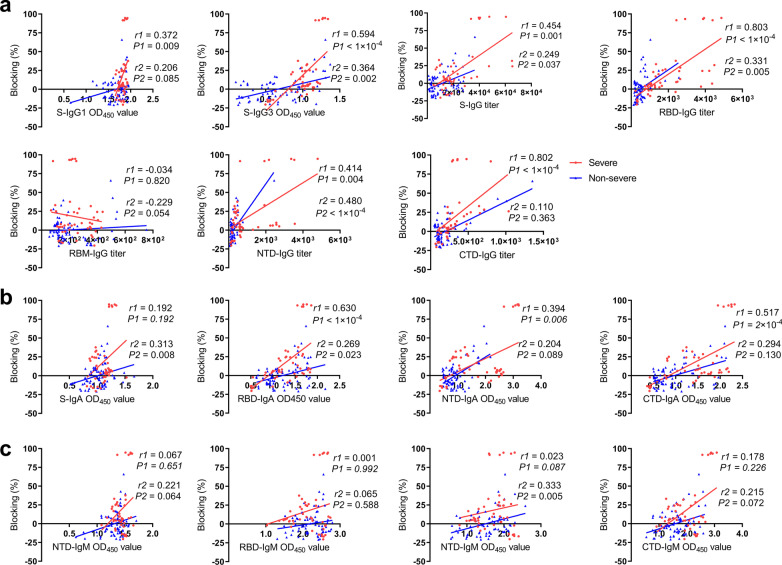
Correlations between blocking percentage and antibody response in severe group and non-severe group. Correlation of blocking percentage with SARS-CoV-2 specific IgG (**a**), IgA (**b**), and IgM (**c**). The correlations were determined by the Spearman rank method, *P* values < 0.05 and *r* > 0.3 or < − 0.3 were considered statistically significant. Red dots, *r*1 and *P*1 represent sample from severe cases; blue dots, *r*2 and *P*2 represent samples from non-severe cases

To examine whether the serum could result in antiviral activity, we next detected whether the serum antibody could block SARS-CoV-2 RBD to bind the ACE2 receptor, which will exert potential neutralizing activity of SARS-CoV-2 in an infected patient. In severe group, the blocking percentages ranged from − 20.4% to 94.7% (median 7.3%), which was significantly higher than non-severe group (− 20.8–65.9%, median − 2.7%, *P* = 5 × 10^–4^) (Fig. 2a). While only some samples exhibited a good inhibitory effect, others did not block RBD-ACE2 engagement and seemed the ACE-2 binding-enhanced signal. Obviously, the severe group showed a higher positive blocking rate (75.0%) than the non-severe group (42.3%) (Fig. 2b). Positive correlations were found between antibody titers and blocking percentage. In severe group, the blocking percentage were positively correlated with S-IgG1 (*r* = 0.372, *P* = 0.009), S-IgG3 (*r* = 0.594, *P* < 1 × 10^–4^), S-IgG titer (*r* = 0.454, *P* = 0.001), NTD-IgG titer (*r* = 0.414, *P* = 0.004), RBD-IgA (*r* = 0.603, *P* < 1 × 10^–4^), NTD-IgA (*r* = 0.394, *P* = 0.006) and CTD-IgA (*r* = 0.517, *P* = 2 × 10^–4^), the blocking percentage were especially strongly correlated with RBD-IgG titer (*r* = 0.803, *P* < 1 × 10^–4^) and CTD-IgG titer (*r* = 0.802, *P* < 1 × 10^–4^) (Fig. 3). In non-severe group, S-IgG3 (*r* = 0.364, *P* = 0.002), RBD-IgG titer (*r* = 0.331, *P* = 0.005), NTD-IgG titer (*r* = 0.480, *P* < 1 × 10^–4^), S-IgA (*r* = 0.313, *P* = 0.008), and NTD-IgM (*r* = 0.333, *P* = 0.005) were positively associated with blocking percentage.

### Serum antibody binding titers with Fc receptors

**Fig. 4:**
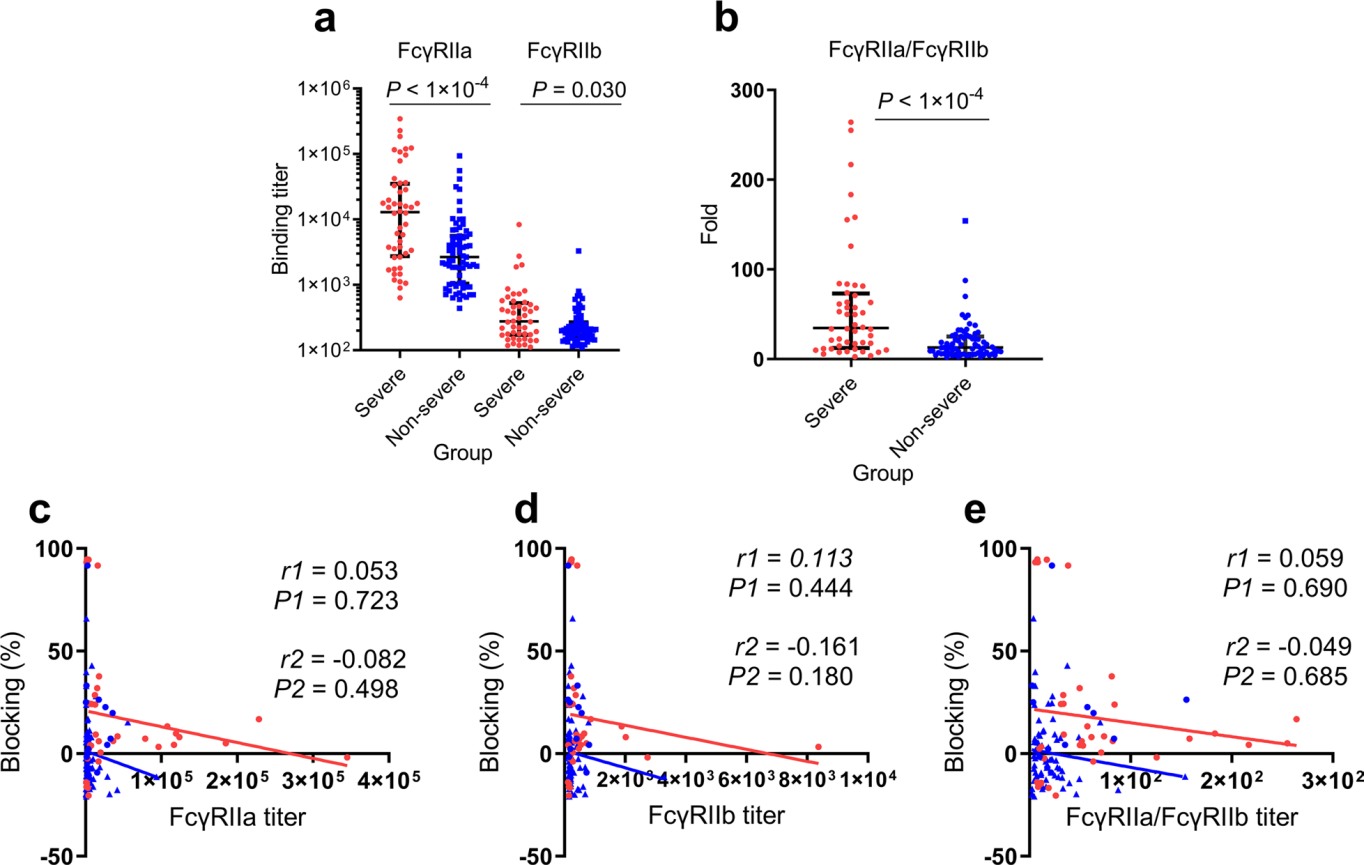
Comparison of binding titers with FcγRIIa and FcγIIb in serum samples and the correlations with blocking rate. **a** Binding titers of serum samples to FcγRIIa and FcγRIIb in severe cases and non-severe cases. **b** Comparison of specific ratio of FcγRIIa/FcγRIIb in severe group and non-severe group. Mann–Whitney U test was used to compare differences between the two groups. Correlations between blocking percentage and FcγRIIa titer (**c**), FcγRIIb titer (**d**), FcγRIIa/ FcγRIIb (**e**). Red dots, *r*1 and *P*1 represent sample from severe cases; blue dots, *r2* and *P2* represent samples from non-severe cases. *P* values < 0.05 and* r* > 0.3 or < − 0.3 were considered statistically significant

To detect whether the difference of serum samples in inhibition or enhancement RBD binding with ACE2 was non-specifically induced by Fc function of serum antibodies, we examined the binding activity of serum sample to Fc receptors, which included an activating receptor FcγRIIa and an inhibitory receptor FcγRIIb. The binding titer of serum antibody to FcγRIIa ranged from 635 to 345,005 (median 12,953) in a severe group and 437–94,649 (median 2,653) in a non-severe group, while binding titers to FcγRIIb ranged from 111 to 8,375 (median 276) in severe group and 111 to 3,287 (median 204) in non-severe group. Notably, both FcγRIIa and FcγRIIb binding titer were significantly higher in a severe group than the non-severe group (*P* < 1 × 10^–4^ and *P* = 0.030, respectively) (Fig. 4a). However, no correlation was found between the blocking rate and FcγRIIa titer in both severe group (*r* = 0.053, *P* = 0.723) and non-severe group (*r* = − 0.082, *P* = 0.498) (Fig. 4c), nor was the correlation between blocking rate and FcγRIIb titer in a severe group (*r* = 0.113, *P* = 0.444) and non-severe group (*r* = − 0.161, *P* = 0.180) (Fig. 4d). In addition, we performed an analysis using the ratio of FcγRIIa and FcγRIIb binding titers in severe group and non-severe group. Consistent with the binding titers in the separate groups, this ratio in a severe group is significantly higher than the non-severe group (*P* < 1 × 10^–4^) (Fig. 4b), and no correlation with ACE2-blocking was found ((Fig. 4e), indicating severe group’s Igs FcγR-binding activity is much stronger.

### Differential expression profiles of cytokines in severe and non-severe case

**Fig. 5:**
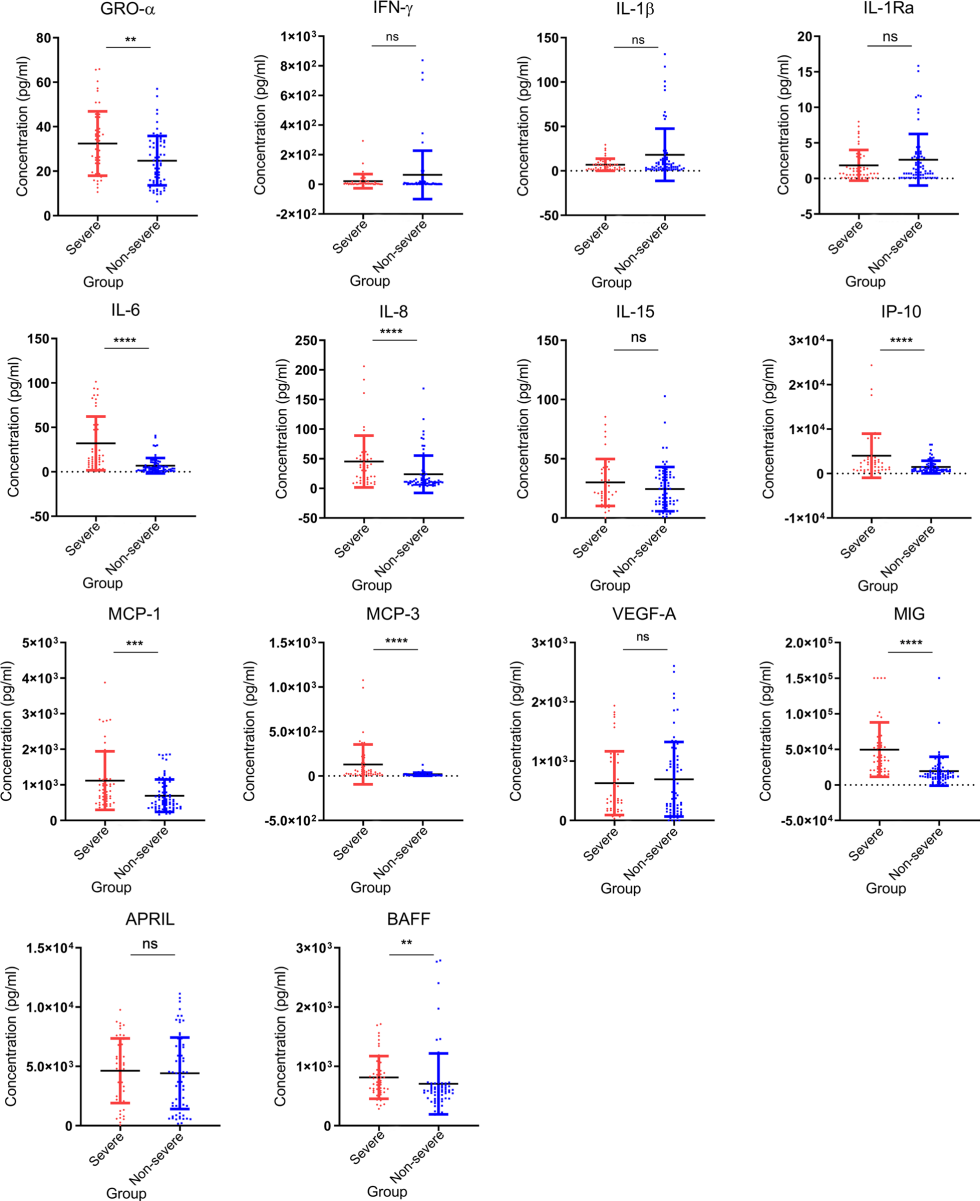
Comparison of serum cytokine/chemokine concentrations between the severe and non-severe COVID-19 cases. Samples from severe (*n* = 45) and non-severe COVID-19 cases (*n* = 74) collected during hospitalization were used for measuring the concentrations of 12 cytokines and chemokine. Values were presented in units of pg/ml. Red dots represent sample from severe cases, blue dots represent samples from non-severe cases. Mann–Whitney U test was used to compare cytokine levels between two groups. Significances were marked as follows: *P* < 0.05 (*), *P* < 0.01(**), *P* < 0.001 (***), and *P* < 0.0001 (****), respectively

To assess other immune factors in blood samples, we continued to analyze the profile of cytokines in COVID-19 patients. An elevated level of nine pro- and anti-inflammatory cytokines were observed in the severe cases as compared with that of the non-severe cases. For severe group, IL-6, IL-8, IP-10, MCP-3, and MIG showed the most significant elevation (*P* < 1 × 10^–4^), followed by MCP-1 (*P* = 3 × 10^–4^), GRO-α (*P* = 0.006) and BAFF (*P* = 0.003). Differences of IFN-γ, IL-1β, IL-1Ra, IL-15, VEGF-A, and APRIL between two groups were not statistically significant (*P* > 0.05) (Fig. 5). These results suggest that significantly higher inflammation responses in a severe group than the non-severe group infected by SARS-CoV-2.

### Specificity and correlation of antibody responses in severe and non-severe group

**Fig. 6:**
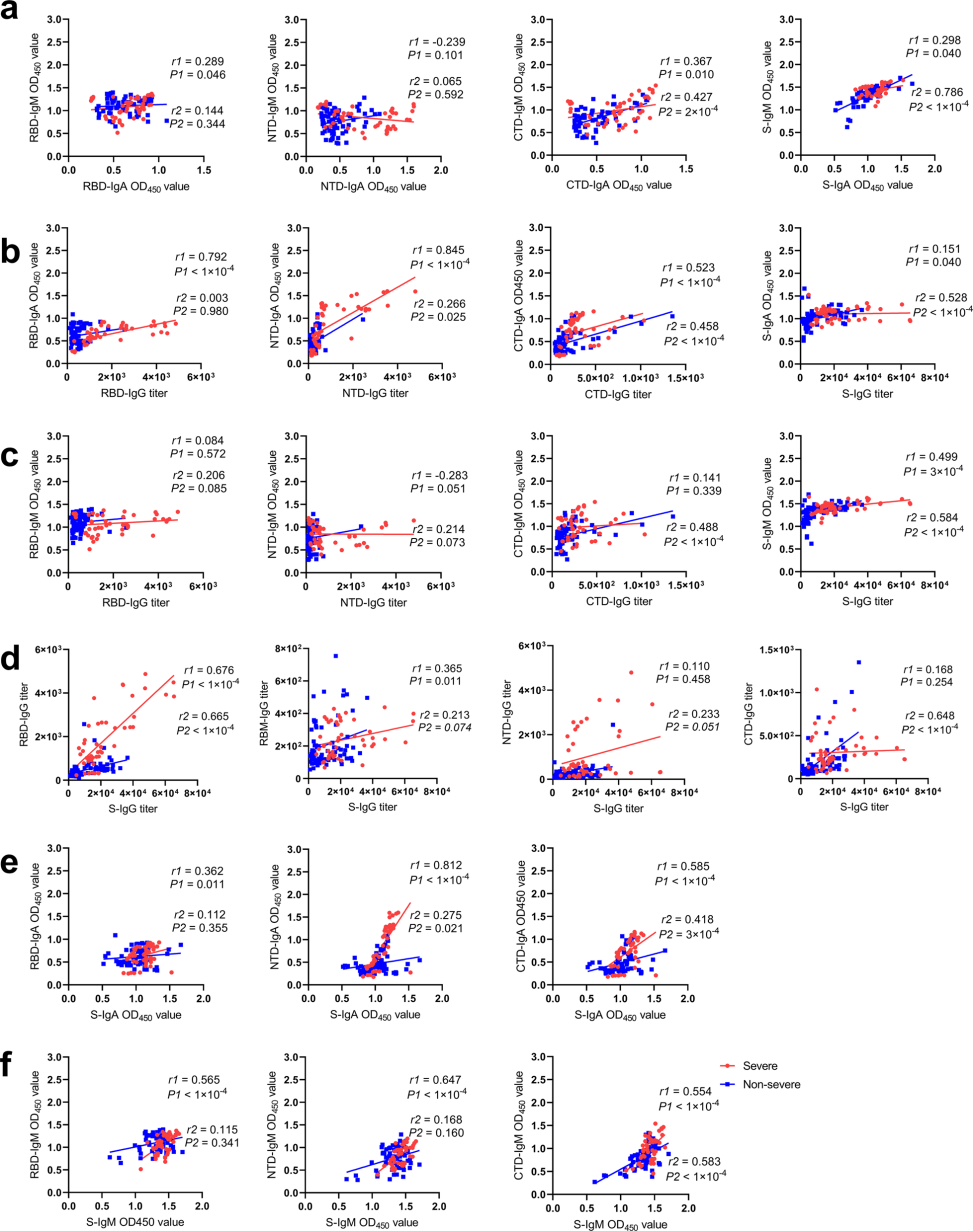
Correlations of antibody isotypes and specific antibodies targeting different antigens. The correlations between antibody level of IgM and IgA targeting S or different S domain (**a**), correlations between IgG and IgA targeting S or different S domain (**b**), correlations between IgG and IgM targeting S or different S domain (**c**), correlation between S-IgG and different S-domain-targeting IgG (**d**), correlation between S-IgA and different S-domain-targeting IgA (**e**), and correlation between S-IgM and different S-domain-targeting IgM (**f**). The correlations were determined by the Spearman rank method,* P* values < 0.05 and *r* > 0.3 or < − 0.3 were considered statistically significant. Red dots, *r*1 and *P*1 represent sample from severe cases; blue dots, *r*2 and *P*2 represent samples from non-severe cases

The results above indicated that the severe group’s antibody levels were much higher than the non-severe group’s antibody levels. To investigate the feature of Ig, we analyzed the correlations of Ig isotypes and IgG titers of different domain targeting antibodies. For correlation analysis between IgA and IgM, positive correlations between CTD-IgA and CTD-IgM were found in both severe group (*r* = 0.367, *P* = 0.010) and non-severe group (*r* = 0.427, *P* = 2 × 10^–4^), whereas positive correlation between S-IgA and S-IgM were found only in the non-severe group (*r* = 0.786, *P* < 1 × 10^–4^) (Fig. 6a). Despite that S-IgG titer was strongly correlated with S-IgA only in the non-severe group (*r* = 0.528, *P* < 1 × 10^–4^), strong correlation between RBD-IgG titer and RBD-IgA titer (*r* = 0.792, *P* < 1 × 10^–4^), between NTD-IgG titer and NTD-IgA titer (*r* = 0.845, *P* < 1 × 10^–4^) were found in the severe group, while correlation between CTD-IgG titer and CTD-IgA was found in both severe group (*r* = 0.523, *P* < 1 × 10^–4^) and non-severe group (*r* = 0.458, *P* < 1 × 10^–4^) (Fig. 6b). Meanwhile, a significant correlation between S-IgG and S-IgM was also observed in both the severe group (*r* = 0.499, *P* = 3 × 10^–4^) and non-severe group (*r* = 0.584, *P* < 1 × 10^–4^), and CTD-IgG titer was correlated with CTD-IgM in the non-severe group only (*r* = 0.488, *P* < 2 × 10^–4^) (Fig. 6c). Further analysis in comparing correlations of antibody belonging to the same Ig isotype (IgG, IgA, and IgM) that targeting S protein or different S domain was showed in Fig. 6d–f. Notably, RBD-IgG titer was strongly correlated with S-IgG titer both in a severe group (*r* = 0.676, *P* < 1 × 10^–4^) and non-severe group (*r* = 0.665, *P* < 1 × 10^–4^). Besides, RBM-IgG was positively correlated with S-IgG in a severe group (*r* = 0.365, *P* = 0.011), while CTD-IgG was positively correlated with S-IgG in a non-severe group (*r* = 0.648, *P* < 1 × 10^–4^) (Fig. 6e). In addition, correlation between S-IgA and NTD-IgA (*r* = 0.812, *P* < 1 × 10^–4^) were much stronger than correlation between S-IgA and RBD-IgA (*r* = 0.362, *P* = 0.011) and correlation between S-IgA and CTD-IgA (*r* = 0.585, *P* < 1 × 10^–4^) in the severe group. While in the non-severe group, correlation between S-IgA and CTD-IgA was the highest (*r* = 0.418, *P* = 3 × 10^–4^) (Fig. 6f). S-IgM showed a positive correlation with RBD-IgM (*r* = 0.565, *P* < 1 × 10^–4^), NTD-IgM (*r* = 0.647, *P* < 1 × 10^–4^), and CTD-IgM (*r* = 0.554, *P* < 1 × 10^–4^) in the severe group, while in the non-severe group, positive correlation was only found between S-IgM and CTD-IgM (*r* = 0.583, *P* < 1 × 10^–4^). The correlation differences of antibody response were summarized in Table 2. Together, different preference of targeting epitope by the three Ig isotypes in severe and non-severe group was observed. The CTD domain was frequently targeted by antibodies in the non-severe group, whiles the RBD domain and NTD-domain were the main target on S for SARS-CoV-2 specific IgG and IgA in the severe group.

**Table 2:**
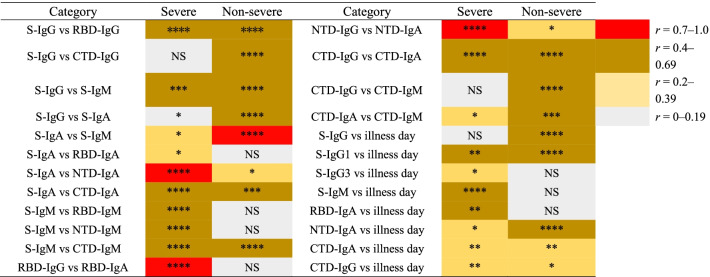
Brief summary of direct antibody responses in correlation analyses between severe and non-severe COVID-19 infections

The colors are marked according to the absolute spearman *r* value, the darker red color represented the higher *r* value. Significances were marked as follows: *P* < 0.05 (*), *P* < 0.01(**), *P* < 0.001 (***), and *P* < 0.0001 (****), respectively, while “NS” represented no significance

### Correlations between antibody responses and days after symptoms onsets in two groups

**Fig. 7:**
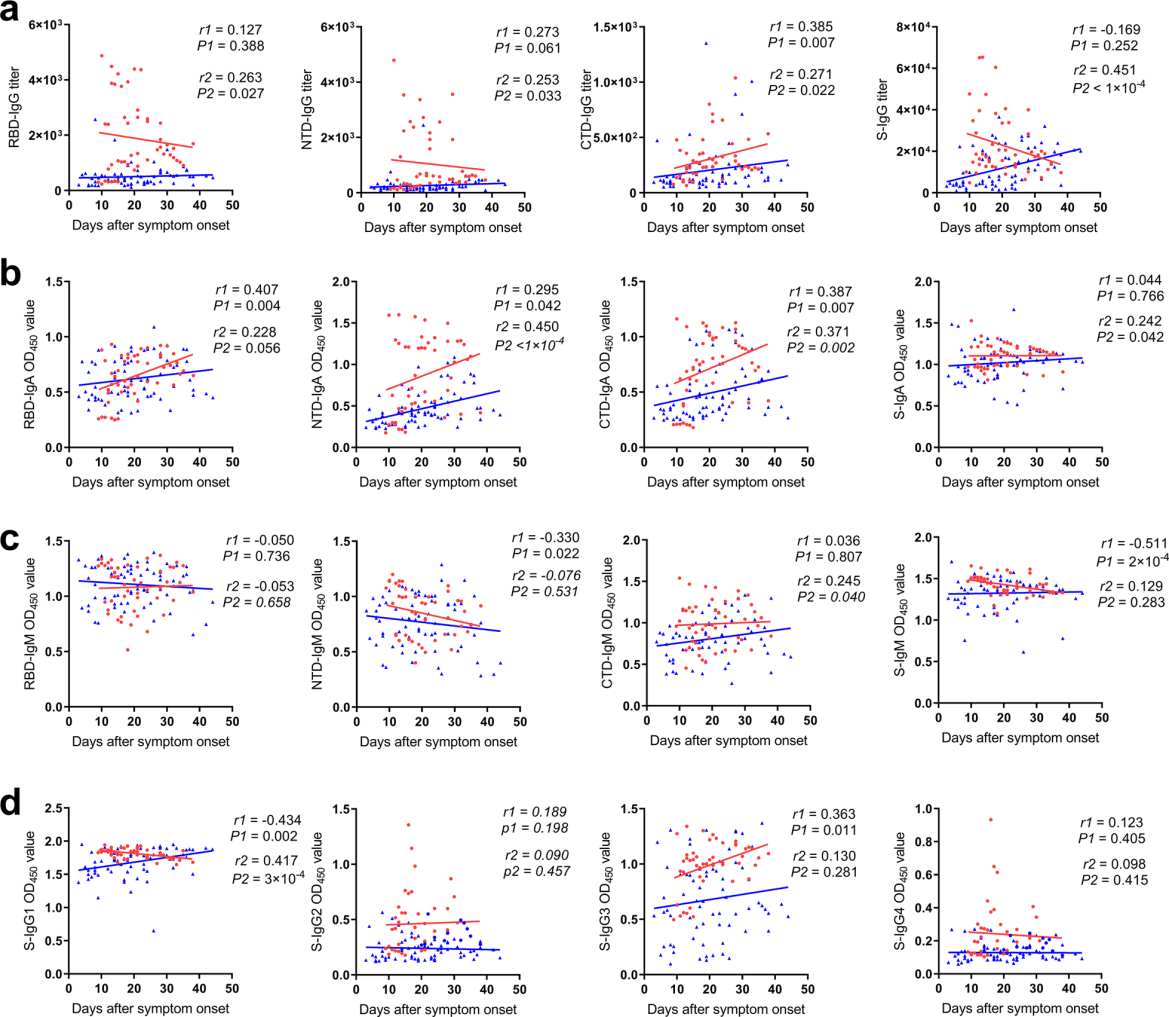
Correlations of specific antibody responses and illness day. The correlations between days after symptom onset and IgG titers (**a**), IgA levels (**b**), IgM levels (**c**), and IgG subclasses (**d**) were determined by the spearman rank method, *P* values < 0.05 and *r* > 0.3 or < − 0.3 were considered statistically significant. Red dots, *r*1 and *P*1 represent sample from severe cases; blue dots, *r*2 and *P*2 represent samples from non-severe cases

In analyzing the specificity of antibody responses, the main targeting domain for SARS-CoV-2’ S spike specific antibody varies between the two groups. We continued to investigate the correlations between antibody responses and days since symptom onset (illness day) against the two groups (Table 2). In terms of the non-severe group, significantly increased S-IgG titer can only be detected in later days after symptom onsets (*r* = 0.451, *P* < 1 × 10^–4^) (Fig. 7a). Similarly, in the severe group, accompanied with more time after symptom onsets, CTD-IgG titers maintained at a higher level with symptom lasted (*r* = 0.385, *P* = 0.007), while that correlation with RBD-IgG titers or NTD-IgG titers were not significant (*r* < 0.3) (Fig. 7a). Besides, RBD-IgA (*r* = 0.407, *P* = 0.004) and CTD-IgA (*r* = 0.387, *P* = 0.007) in the severe group, NTD-IgA (*r* = 0.450, *P* < 1 × 10^–4^) and CTD-IgA (*r* = 0.371, *P* = 0.002) in the non-severe group showed positively correlation with illness day (Fig. 7b). In addition, NTD-IgM (*r* = − 0.330, *P* = 0.022) and S-IgM (*r* = − 0.511, *P* = 2 × 10^–4^) were negatively correlated with days after symptom onset in the severe group only (Fig. 7c). Notably, S-IgG1 was negatively associated with illness day in a severe group (*r* = − 0.434, *P* = 0.002), while S-IgG3 in a severe group (*r* = 0.363, *P* = 0.011) and S-IgG1 (*r* = 0.417, *P* = 3 × 10^–4^) in the non-severe group was positively associated with days after symptom onset (Fig. 7d). These results suggest that different antibody dynamics between the severe group and non-severe group induced by SARS-CoV-2 infection.

## Discussion

In this study, we investigated clinical features and antibody response, including antibody level, specificity, Ig isotypes and IgG subtypes, ACE2 competitive antibody function, FcγR-binding activity, and a panel of 14 cytokine levels of COVID-19 patients. We also sought to understand the clinical and immune response characteristics of severe SARS-CoV-2 and non-severe patients. Finally, we determined the different signatures in clinical and antibody responses in these two groups.

Consistent with what was previously reported [22], we observed that LDH, D-dimer, CRP, the concentration of prothrombin, TBA, r-glutaminase, adenosine deaminase in severe group were significantly higher than a non-severe group. We also found a significantly lower proportion of lymphocytes and higher neutrophil count and percentage in the severe group than a non-severe group. These results indicated these markers found in the laboratory could be used for predicting severe cases, and should be paid more attention to during treatment.

In antibody response, similar to a previous study [23], we observed a significantly higher titer of S-specific IgA, IgG, and IgM in a severe group than that of non-severe patients. We also observed significant positive correlations of S-IgM and S-IgG in the two groups. However, the correlations of S-IgA and S-IgG, S-IgA and S-IgM, were just shown in the non-severe group. Indeed, the S- specific antibody isotype switch might be different between these two groups. Meanwhile, IgA and IgG, showed no association with illness days during the hospitalization in severe group, which was different from that in non-severe group. Similar to the previous reports [8, 24], it is likely that the production of antibody is faster and stronger in severe group, and IgA and IgG antibody maintained better than non-severe group.

It is reported that IgG subclasses were negatively correlated to viral load [23]. In our study, we found that S-IgG1 and S-IgG3 were majority subclass IgG induced by SARS-CoV-2 infection. Furthermore, negative correlation between IgG1 and days after symptom onset, positive correlation between IgG3 and days after symptom onset in severe group were found in our analysis. While in non-severe group, we only found positive correlation between IgG1 and illness days, and no association between IgG3 and illness days. One possibility might be that in the early illness stage, the higher IgG1 response accompanied with COVID-19 symptoms. In the later stage in the severe group, level change of IgG1 and IgG3 seemed to produce unidentified antibody response’s effect against illness severity. As we know, the IgG1 and IgG3 were the main antibody that could induce antibody-dependent cell-mediated cytotoxicity (ADCC) due to their high affinity with FcγRs and were helpful for the elimination of viruses. We also found significantly higher IgG1 and IgG3 responses in the severe group. Moreover, IgG1 and IgG3 in the severe group, IgG3 in the non-severe group showed a significant correlation with RBD-ACE2 blocking rate, which was similar to the Luo et al. study that S-specific IgG1 and IgG3 were associated with disease severity and were correlated with reduced virus load in nasopharyngeal swab [23]. Furthermore, we found significantly higher binding titer of FcγRIIa and FcγRIIb in the severe group, as well as the ratio of FcγRIIa/FcγRIIb. Thus, it is worth investigating whether IgG subclasses especially IgG1 and IgG3 and binding with RcγRs exerts different antiviral activity in the progress of SARS-CoV-2 infection and leads to different severity of the disease.

Plasma anti-SARS-CoV-2 spike protein and receptor-binding domain IgG were helpful for virus neutralization by blocking the interaction between RBD and the virus receptor AEC2 [25, 26]. Our study showed that the SARS-CoV-2 specific antibody consisted of RBD- targeting antibody and high titers of NTD- and CTD- targeting antibody, resulting in correlation with blocking rate, which indicated the important function of NTD- and CTD-reactive antibody in serum. Positive correlations between RBD-targeting antibody titers and serum blocking rate of RBD-ACE2 were found in both groups. In addition, NTD-IgG was also associated with blocking rate in both the severe and non-severe groups, and CTD-IgG in the severe group significantly correlated with blocking rate. However, the receptor binding motif (RBM), did not show a significant correlation with the blocking rate. Based on the RBM-IgG titers were much lower than that of RBD (123–4,871), NTD (68–4,795), CTD (50–1,353), our results indicated that the linear epitope of RBM was less frequently targeted and was not a good choice of immunogen.

In addition, although all of the specific IgG levels were significantly higher in the severe group than the non-severe group, only NTD-IgA, CTD-IgA, and CTD-IgM showed a significant higher level in the severe group than in the non-severe group. Notably, CTD seemed to be an important target on S protein in the non-severe group because positive correlations between S-IgG and CTD-IgG, S-IgA and CTD-IgA, S-IgM and CTD-IgM were observed in the non-severe group. Furthermore, it seemed that the epitope targeted by the three Ig isotypes varied between the two groups. For the Igs isotype analysis, RBD-IgG showed the highest correlation with S-IgG in both the severe and non-severe group. NTD-IgA showed the highest correlation with S-IgA in severe group, whereas CTD-IgA correlated with S-IgA in the non-severe group. And only CTD-IgM showed positive correlation with S-IgM in the non-severe group, contrast to the severe group where RBD-IgM, NTD-IgM, and CTD-IgM were all correlated with S-IgM. Several studies have reported that the combined immunogens of different domain of S protein exhibited more robust and stable immunogenicity and higher neutralization potency [13, 14, 27–29]. Therefore, in the future, more attention should be paid to detecting and isolating NTD-directed or CTD-directed neutralizing antibodies, and immunogens may not only just be based on RBD but also based on other domains of S such as NTD.

Previous studies have shown that elevated levels of proinflammatory cytokines, such as IL-1β, IL-1Ra, IL-6, IL-8, IL-9, IL-10, IFN-γ, IP-10, MCP-1 and MCP-3 are associated with severe lung injury and adverse outcomes in SARS-CoV or MERS CoV infection, and IP-10, IL-10 and IL-6 could anticipate subsequent clinical progression [20, 21, 30, 31]. Our results also showed that the IL-6, IL-8, IP-10, MCP-1, MCP-3, and MIG were significantly different between severe cases and non-severe cases, suggesting that the magnitude of these cytokines is associated with the disease severity, which reflects dysregulated immune response. Therefore, the combinatorial analysis of clinical classification with serum cytokines can contribute to better evaluating the severity of COVID-19 and optimizing the therapeutic strategies. Besides, we found significantly higher BAFF levels in the severe COVID-19 group than the non-severe group, indicating robust activation of B cell response associated with BAFF in severe COVID-19 patients when corresponding to overall higher antibody responses in severe group. Since BAFF and APRIL, the agents associated with B cell activation and maturation have been reported to play roles in the pathogenesis of HIV-1 and HCV [32, 33]; next, we should explore the functional characteristics of BAFF during SARS-CoV-2 infection.

Clinical and demographic features of COVID-19 patients have recently been reported [4, 34, 35], and some immunological features were subsequently reported [6]. Characterization of the clinical and immune response of COVID-19 patients, such as our now study about clinical features and antibody responses signatures of severe SARS-CoV-2 and non-severe patients, is still valuable to understand SARS-CoV-2 infection. Although the relatively small sample size was one of the limitations in the current study, we still detected dysregulated antibody responses, hyperinflammation and lymphopenia due to the severity infected by SARS-CoV-2. In the future, more sample sizes should be added, and the relationship of antibody response and other clinical features, the roles of Igs, Fc effector function, influences of uncertain cytokines in COVID-19 patients should be further investigated in larger cohorts.

## Conclusions

In this study, we found diverse clinical and antibody response between the COVID-19 severe group and non-severe group, mainly on antibody isotype and IgG titers, antibody specificity and dynamics, RBD-ACE2 blocking activity, FcγR binding capacity, B cell activation factor, and cytokines. Finding the specific Igs, Fc effects, and influences of B cell-activating cytokines in COVID-19 patients will contributed to future therapeutic and preventive measures development.

## Supplementary Information

**Additional file 1: Table S1.** Clinical characteristics between severe and non-severe COVID-19 patients

**Additional file 2: Table S2.** Proteins used in this study.

## Data Availability

All data generated or analyzed during this study are included in this published article or the Additional files.

## Abbreviations

S: Spike
RBD: Receptor Binding Domain
RBM: Receptor binding motif
NTD: N-terminal Domain
CTD: C-terminal Domain
