# Supplementary material for: Clinical and antibody characteristics reveal diverse signatures of severe and non-severe SARS-CoV-2 patients
Source: Infect Dis Poverty. 2022 Feb 2;11:15. doi: 10.1186/s40249-022-00940-w (PMC8809634; doi:10.1186/s40249-022-00940-w)
Supplement: Supplementary file 1 — Additional file 1: Table S1. Clinical characteristics between severe and non-severe COVID-19 patients [file 40249_2022_940_MOESM1_ESM.docx]

**Table S1**. Clinical characteristics between severe and non-severe COVID-19 patients

|  | | | | |
| --- | --- | --- | --- | --- |
| Characteristics | All patients (*n* = 37) | Severe (*n* = 9) | Non-severe (*n* = 28) | *P* value |
| Age | 53.50 (48.50–66.00) | 59.00 (50.00–69.50) | 52.00 (47.00–66.00) | 0.1998 |
| ≤ 50 years, *n* (%) | 11 (29.7%) | 2 (22.2%) | 9 (32.1%) | 0.6946 |
| > 50 years, *n* (%) | 26 (70.3%) | 7 (77.8%) | 19 (67.9%) |  |
| Gender |  |  |  | > 0.9999 |
| Female, *n* (%) | 21 (56.8%) | 5 (55.6%) | 16 (57.1%) |  |
| Male, *n* (%) | 16 (43.2%) | 4 (44.4%) | 12 (42.9%) |  |
| Signs and symptoms |  |  |  |  |
| High temperature (°C) | 37.80 (37.40–38.50) | 38.80 (38.55–39.05) | 37.50 (37.30–38.05) | < 0.0001 |
| Cough, *n* (%) | 34 (91.9%) | 9 (100%) | 25 (89.3%) | 0.5622 |
| Fatigue, *n* (%) | 33 (89.2%) | 9 (100%) | 24 (85.7%) | 0.5536 |
| Sore throat, *n* (%) | 18 (48.6%) | 7 (77.8%) | 11 (39.28) | 0.0625 |
| Chest tightness, *n* (%) | 23 (62.2%) | 9 (100%) | 14 (50%) | 0.0071 |
| Difficulty breathing, *n* (%) | 14 (37.8%) | 8 (88.9%) | 6 (21.4%) | 0.0006 |
| Nausea or vomiting, *n* (%) | 11 (29.7%) | 7 (77.8%) | 4 (14.3%) | 0.0009 |
| Diarrhea, *n* (%) | 12 (32.4%) | 6 (66.7%) | 6 (21.4%) | 0.0355 |
| Myalgia, *n* (%) | 9 (24.3%) | 5 (55.6%) | 4 (14.3%) | 0.0231 |
| Complication |  |  |  |  |
| Diabetes, *n* (%) | 4 (10.8%) | 4 (44.4%) | 0 | 0.0019 |
| Hypertension, *n* (%) | 6 (16.2%) | 3 (33.3%) | 3 (10.7%) | 0.1404 |
| Coronary heart disease, *n* (%) | 1 (2.7%) | 0 | 1 (3.6%) | > 0.9999 |
| Chronic hepatitis, *n* (%) | 4 (10.8%) | 1 (11.1%) | 3 (10.7%) | > 0.9999 |
| Chronic bronchitis, *n* (%) | 2 (5.4%) | 0 | 2 (7.1%) | > 0.9999 |
| Uremia, *n* (%) | 1 (2.7%) | 1 (11.1%) | 0 | 0.2432 |
| Epidemiological history, *n* (%) |  |  |  |  |
| Wuhan's touched history, *n* (%) | 11 (29.7%) | 4 (44.4%) | 7 (25.0% ) | 0.4038 |
| Wuhan's untouched history, *n* (%) | 26 (70.3%) | 5 (55.6%) | 21 (75.0%) |  |
