# Supplementary material for: Clinical and antibody characteristics reveal diverse signatures of severe and non-severe SARS-CoV-2 patients
Source: Infect Dis Poverty. 2022 Feb 2;11:15. doi: 10.1186/s40249-022-00940-w (PMC8809634; doi:10.1186/s40249-022-00940-w)
Supplement: Supplementary file 2 — Additional file 2: Table S2. Proteins used in this study. [file 40249_2022_940_MOESM2_ESM.docx]

**Table S2**. Proteins used in this study

| Name | Cat# | Company |
| --- | --- | --- |
| SARS-CoV-2 Spike Protein (S ectodomain) | 40589-VO8B1 | Sino Biological |
| SARS-CoV-2 Spike RBD | 40592-V08B | Sino Biological |
| SARS-CoV-2 RBM | Customized | Sino Biological |
| SARS-CoV-2 NTD | DRA45 | Novoprotein |
| SARS-CoV-2 CTD | DRA46 | Novoprotein |
| Human ACE2 protein | C419 | Novoprotein |
| FcγRⅡa | CS35 | Novoprotein |
| FcγRⅡb | CS444 | Novoprotein |
